# Lumbar Spine Osteomyelitis After Permanent Spinal Cord Stimulator Implantation: A Case Report

**DOI:** 10.7759/cureus.30967

**Published:** 2022-11-01

**Authors:** Gurtej Bajaj, Russell Bell, Ben Silverman, Sonika Seth

**Affiliations:** 1 Physical Medicine and Rehabilitation, University of Pennsylvania, Philadelphia, USA; 2 Anesthesiology, University of Pennsylvania, Philadelphia, USA

**Keywords:** pain medicine, case report, explant, vertebral osteomyelitis, spinal cord stimulator

## Abstract

A spinal cord stimulator (SCS) is an intervention that has become increasingly popular due to its efficacy in treating pain. With the increasing number of SCSs implanted annually, there has been an equal increase in complications, which include infections. We present a patient who underwent an uncomplicated permanent placement of SCS and later developed worsening back pain, weakness, and fever after a mechanical fall and was subsequently found to have vertebral osteomyelitis without an identifiable infection source. While no source or definitive pathogen was discovered, if there is a concern for osteomyelitis radiographically, even in an uncommon situation when medical workup returns inconclusive, explant of the SCS is warranted.

## Introduction

A spinal cord stimulator (SCS) is an intervention that has become increasingly popular due to its efficacy in treating pain. It is currently approved by the FDA for the treatment of chronic pain of the trunk and limbs, failed back surgery syndrome, chronic regional pain syndrome, and pain from peripheral vascular disease. Over 50,000 SCSs are implanted annually, and with this increase in number, there has been an equal increase in complications [1]. The overall infection rate after SCS placement has been reported to range from about 3 to 10% and most commonly involves the superficial skin and subcutaneous tissue adjacent to the surgical site [2,3]. A more serious and uncommon infection complication is vertebral osteomyelitis which can occur either by hematogenous spread from a distant site, direct inoculation, or contiguous spread from infected tissue [4]. While a source for osteomyelitis can usually be found, it is perplexing when this form of infection is seen without an identifiable cause.

In this case report, we provide a scenario of a patient who underwent an uncomplicated permanent placement of SCS and later developed worsening back pain, weakness, and fever after a mechanical fall and was subsequently found to have vertebral osteomyelitis without an identifiable infection source.

## Case presentation

**Figure 1:**
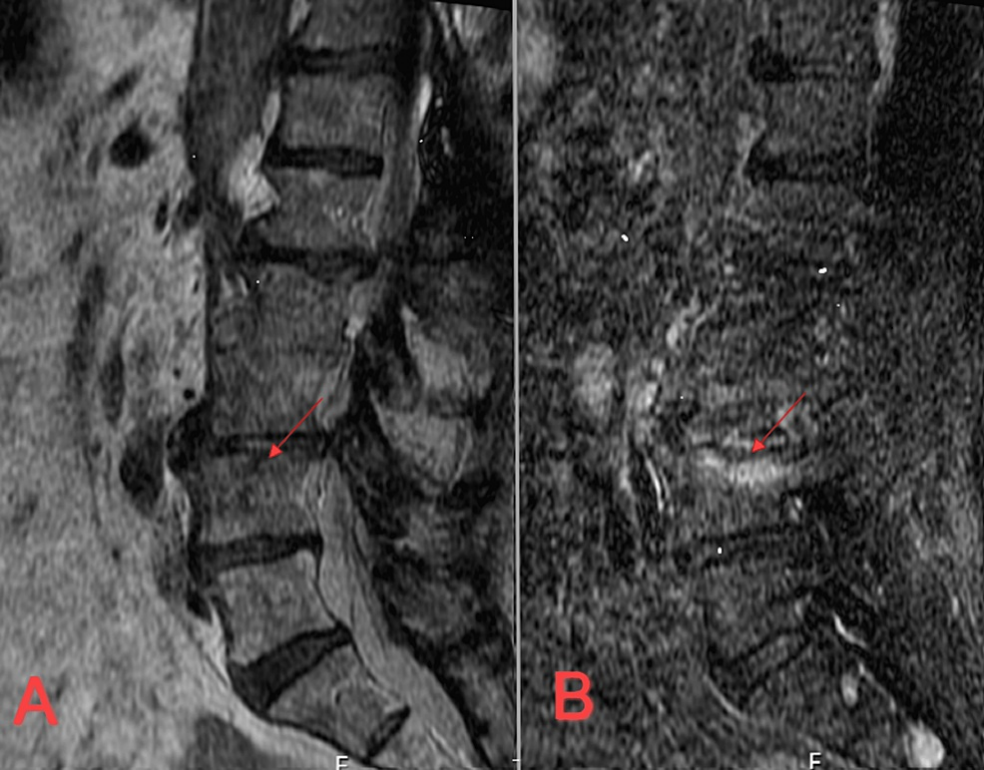
A) a sagittal T2-weighted image of the lumbar spine and the arrow indicates site of osteomyelitis at the level of L3-L4. B) is a corresponding STIR sequence noting ongoing boney infection. STIR: short inversion time inversion recovery

An 87-year-old male with a significant past medical history of cervical laminectomy and fusion in 2021 and L1-L2 laminectomy in 2019 presented to the pain clinic with chronic right axial lower back pain. He has been having pain for the last seven years without alleviation with surgical interventions. On presentation, he was taking oxycodone 10 mg three times a day and gabapentin 300 mg twice a day. Due to the chronicity of his low back pain, a joint decision was made for SCS placement. On June 2022, percutaneous trial leads were placed with notable success in decreasing pain levels such that he did not need a cane for ambulation and was able to decrease the use of oxycodone. In July 2022, the patient underwent permanent SCS placement. Before the procedure, T12 - L1 interspace was identified. The surgical site was cleaned with chlorhexidine gluconate, followed by a sterile adhesive dressing placement and a total body surgical drape. After the surgical incision, insertion of the Touhy needle, and removal of its stylet, the Boston Scientific spinal cord stimulating lead was advanced through the Tuohy needle until the tip of the stimulating lead was aligned with the inferior endplate of the T7 vertebral body. The pulse generator site was identified, marked, and created, with subsequent usage of a tunneling tool to facilitate the connection of the SCS leads to the generator. Both surgical sites were washed thoroughly with saline and antibiotics before being sutured. Six days later, the patient had a mechanical fall where he sustained a laceration of the forehead that required stitches and was subsequently discharged home. He was seen back in the pain clinic, where his SCS leads were programmed and dressings were changed. There was no concern for infection at the surgical sites. Unfortunately, about one month later, he presented to the emergency department with worsening back pain, new right leg weakness with associated leukocytosis, elevated inflammatory markers, and fever. A CT lumbar showed fluid collection L1-4 through which the spinal stimulator lead travels, and subsequent lumbar MRI demonstrated signs of discitis-osteomyelitis at L3-L4 level (Figure 1).

Blood cultures were negative for growth and CT-guided biopsy of the L3-L4 disc space was also negative for both gram stain and culture. It was then recommended by Infectious Disease to treat the patient with six weeks of intravenous vancomycin and ceftriaxone upon discharge. When seen back in the pain clinic days later, it was decided that given the radiological findings and high suspicion this was a true infection, explant of the SCS was necessary and would be facilitated by neurosurgery.

## Discussion

Complications of SCS placement have been reported at a rate of 30-40% which include lead migration, lead fracture, and dural puncture [5,6]. While infection risk is not the most common complication, it may require the removal of implanted hardware that consequently will negate the therapeutic pain relief provided by SCS. As is the nature of any implanted medical device and penetration of the skin, bacterial colonization and invasion are always inherent risks, and prolonged procedural time has been shown to increase the risk of postoperative infection [6]. The generator pocket is the most common infectious site [7]. While a superficial infection of the battery does not necessarily require explantation, a deeper infection yields such a justification [7,8].

The diagnosis of vertebral osteomyelitis can be made by a combination of MRI, the imaging modality of choice, blood cultures, and/or percutaneous biopsy of the area of concern [9-11]. When blood cultures return negative, percutaneous biopsy of the infected vertebra should be considered to determine the causative pathogen. However, in cases when blood cultures are negative, biopsy cultures have been shown only to be positive 43-78% of the time [10]. In such cases where osteomyelitis is suspected but blood cultures and repeat biopsies are negative, the diagnosis of osteomyelitis can be inferred and should be empirically treated [12]. In our case, a second biopsy was not pursued, but enough concern for osteomyelitis was present that the patient was started empirically on intravenous antibiotics.

Intravenous drug use makes one more susceptible to osteomyelitis due to the hematogenous spread of pathogens. A case report by Chien et al. describes a 56-year-old male with a previous history of intravenous drug abuse and chronic low back from decompressive laminectomy who developed fevers, chills, and worsening back pain three months after SCS placement. MRI of the lumbar spine revealed osseous destruction of the L1 and L2 vertebrae, and the contiguous intervertebral disc which was concerning for osteomyelitis. Needle aspiration grew invasive *Candida parapsilosis*, a pathogen that can be isolated in non-human sources. The source of the infection was determined to be through intravenous drug use and required an explant of the SCS [13].

In contrast to this case, the patient had no history of intravenous drug use, but he had radiological findings concerning osteomyelitis. The mechanism as to how this occurred is not known. One possible explanation could be that the equipment or field sterilization was breached, and his fall led to osseous inoculation of the lumbar spine and its subsequent downstream effects. A second explanation, which is less likely, could be seeding from the forehead laceration during his fall. However, if this was to occur, hematogenous spread from the grossly infected forehead and positive blood cultures, both of which were not seen.

Currently, there are no consensus guidelines for when SCS can be replanted after its explant was due to an infectious etiology. Interestingly, a case report described a patient whose SCS was removed after notable surgical site dehiscence and required six weeks of intravenous antibiotics. The SCS was replanted one year after its removal, due to patient preference, with similar alleviation of her chronic pain symptoms [14]. Independent of patient preference, The Neurostimulation Appropriateness Consensus Committee recommends reimplantation can take place 90 days after the complete resolution of the infection [15].

## Conclusions

This case report highlights an uncommonly reported situation of a patient who develops vertebral osteomyelitis without a definitive source of infection. When implanting a foreign body, deep infections are a possible risk that requires immediate attention from clinicians. If there is a concern for osteomyelitis radiographically, even in an uncommon situation when a medical workup returns inconclusive, explant of the SCS and administration of antibiotics is warranted. A thorough assessment of infection clearance must be carried out prior to any consideration of re-implant of the SCS.
